# The dose–effect relationships of cigarette and alcohol consumption with depressive symptoms: a multiple-center, cross-sectional study in 5965 Chinese middle-aged and elderly men

**DOI:** 10.1186/s12888-022-04316-0

**Published:** 2022-10-25

**Authors:** Qian Liu, Pei Leng, Yiqun Gu, Xuejun Shang, Yuanzhong Zhou, Huiping Zhang, Liandong Zuo, Guangan Mei, Chengliang Xiong, Tianpeng Wu, Honggang Li

**Affiliations:** 1 Center for Reproductive Medicine, Wuhan Children’s Hospital, Tongji Medical College, Huazhong University of Science and Technology, Wuhan, 430000 China; 2 Institute of Reproductive Health, Tongji Medical College, Huazhong University of Science and Technology, Wuhan, 430030 China; 3 National Health and Family Planning Key Laboratory of Male Reproductive Health, National Research Institute for Family Planning, Beijing, 100000 China; 4 Department of Andrology, School of Medicine, Jinling Hospital, Nanjing University, Nanjing, 210093 China; 5 School of Public Health, Zunyi Medical University, Zunyi, 563000 Guizhou China; 6 Wuhan Tongji Reproductive Medicine Hospital, Wuhan, 563000 China; 7 Guangzhou Women and Children’s Medical Center, Guangzhou, 510000 China; 8 Technical Guidance Institute of Shanxi Province Family Planning Commission, Xi’an, 710000 China; 9 Department of Urology, Renmin Hospital of Wuhan University, Wuhan, 430060 China

**Keywords:** Cigarette, Alcohol, Depressive symptoms, Dose–response relationship, Middle-aged and elderly men, Cigarette cessation

## Abstract

**Background:**

Although association of depressive symptoms with cigarette or alcohol is well documented, the dose–response relationship between them is rarely studied. This study aims to evaluate dose–response relationships of cigarette and alcohol consumption with depressive symptoms in Chinese middle-aged and elderly men, providing evidence to guide cigarette and alcohol control.

**Methods:**

This multiple-center, cross-sectional study including 5965 Chinese men aged 40–79 years was conducted in 2013–2016 in China. Depressive symptoms were evaluated by Beck Depression Inventory-Short Form. History of cigarette smoking and alcohol drinking were collected with a structured questionnaire. Prevalence of depressive symptoms was compared depending on cigarette and alcohol consumption. Adjusted odds ratios (OR) and 95% confidence interval (CI) were estimated by binary logistic regression. Interpolation analysis was applied to test dose–effect relationships.

**Results:**

A parabolic-shaped relationship was observed between cigarette consumption and depressive symptoms. Compared to never smokers, 59.0% (OR = 1.59, 95% CI 1.30–1.94) and 29.0% (OR = 1.29, 95% CI 1.08–1.54) higher odds of depressive symptoms were observed in men smoking < 10 cigarettes/day and 10–20 cigarettes/day, whereas, similar odds of depressive symptoms among men smoking > 20 cigarettes/day (*P* = 0.092). An inverted J-shaped relationship was observed between alcohol consumption and depressive symptoms. Compared to never drinkers, a tendency of higher prevalence of depressive symptoms (OR = 1.16, 95% CI 0.99–1.36) was observed in men drinking < 140 g/week, and similar prevalence was observed in those drinking 140–280 g/week (*P* = 0.920), whereas, 29.4% (OR = 0.71, 95% CI 0.57–0.88) lower odds in men drinking > 280 g/week.

**Conclusions:**

Associations of cigarette smoking and alcohol drinking with depressive symptoms differ with consumption in middle-aged and elderly men. Health-care providers should exercise great caution on depressive symptoms in conducting cigarette and alcohol control.

**Supplementary Information:**

The online version contains supplementary material available at 10.1186/s12888-022-04316-0.

## Background

Depression is a common mental illness in middle-aged and elderly men [1, 2], which reduces their life quality and expectancy, as well as increases risk of mortality and suicide [1–3]. Cigarette smoking and alcohol drinking are common lifestyles in middle-aged and elderly men [4–8]. A positive association between cigarette smoking and depressive symptoms has been consistently documented among both men and women ranging from the adolescents to older people [4–6]. Whereas, association of alcohol drinking with depressive symptoms differs with drinking patterns [7–10]. Regarding mild to moderate drinking, some studies have suggested a protective effect on depressive symptoms [7–9], whereas, others report no association [10]. As for heavy drinking or alcohol use disorder, a positive association with depressive symptoms has been well established in both retrospective and prospective studies [11–13]. However, although the association between depressive symptoms and cigarette smoking or alcohol drinking is well documented, the dose–response relationship between them has not been fully understood.

The association between depressive symptoms and cigarette smoking or alcohol drinking has been implied to differ with certain demographic, social-economic and cultural characteristics of the study population e.g., ethnicity [5, 14–16], age [15, 16], gender [17–20], education, region. Therefore, it’s reasonable to assume that these characteristics of the study population may also affect the dose–response relationships. China has different cigarette smoking and alcohol drinking patterns from Western high-income countries due to genetic and cultural factors [21–24]. Distinct from high prevalence of heavy drinking or alcohol use disorder in Western high-income countries, Chinese drinking pattern is characterized by moderate alcohol intake and a preference for drinking with meal or at social occasions [21–23, 25, 26]. And majority of previous studies were carried out among adolescents or young adults and often conducted a mixed analysis including both men and women [5, 6, 27–30]. The presentation and trajectory of drinking, smoking, and depressive symptoms are different in older adults compared to adolescents and men compared to women [15, 16, 18–20]. In order to produce the most pertinent evidence to guide prevention and intervention efforts, it is important to generate ethnicity-, age- and sex-specific estimates of the associations and dose–response relationships of cigarette smoking and alcohol drinking with depressive symptoms.

Together, due to limitations of the previous studies, i.e., insufficient understanding regarding the dose–response relationships [4–8], and limited evidences in Chinese middle-aged and elderly men [11, 31], a large-scale multi-center study among Chinese middle-aged and elderly men evaluating the dose–response relationships of cigarette smoking or alcohol drinking with depressive symptoms is needed. In this multi-center, cross-sectional study, we compared the prevalence of depressive symptoms among 5965 Chinese middle-aged and elderly men with different cigarette smoking or alcohol drinking status. And we further evaluated the dose–response relationships of cigarette and alcohol consumption with depressive symptoms. Besides, cigarette smoking and alcohol drinking are often concomitant among middle-aged and elderly men, we therefore explored whether interaction exists between cigarette smoking and alcohol drinking regarding associations with depressive symptoms. It was hypothesized that associations of cigarette smoking and alcohol drinking with depressive symptoms would differ with consumption in middle-aged and elderly men.

## Methods

### Study participants

The Chinese Male Aging Study, a nationwide, cross-sectional study, was conducted during a period from June 1, 2013 to August 31, 2016 in six provinces in China (Jiangsu, Guizhou, Shanxi, Hebei, Guangdong, and Hubei) that were selected based on logistic support and to ensure a mixture of geography, socioeconomic status, and lifestyle representative of the entire country. Stratified-random-cluster sampling was performed, the details of which are available elsewhere [32]. Briefly, the population in each province was firstly stratified into five layers according to the administrative level, and one community (urban or rural) was sampled for each layer. Second, random-cluster sampling was used to select one community in each layer of the province. Finally, five communities (urban or rural) were sampled for each province. All men aged 40–79 years in the selected communities were invited to participate. The Montreal Cognitive Assessment was applied for screening of cognitive disorder. Subjects with a total score ≥ 26 were considered suffering from cognitive disorder and excluded from this study.

### Evaluation of depressive symptoms

Depressive symptoms were evaluated by Beck Depression Inventory-Short Form [33, 34]. The questionnaire consists of 13 self-report items that express specific symptoms of depression. Each item contains 4 statements scoring from 0–3. BDI-SF has shown good validity and reliability in Chinese population [33, 34]. A total score 0–4, 5–7 8–15 and ≥ 16 were classified as no, mild, moderate, and severe depressive symptoms, respectively [33]. Participants self-administrated BDI-SF under the assistance of a trained staff during a face-to-face interview.

### Assessment of cigarette smoking and alcohol drinking

Information on demographic characteristics (age, ethnicity, education, residence, occupation, marital status), disease history, cigarette smoking (smoking status, smoking years and daily cigarette consumption), and alcohol drinking (drinking status, drinking years and weekly alcohol consumption) were collected with a structured questionnaire that was completed by the participants [35].

According to smoking status, participants was categorized as never smoker; as current smoker, i.e., men who had smoked cigarettes for ≥ 6 months and were still smoking at the time of interview; or as past smoker, i.e., men who had stopped smoking at least 6 months before the interview [36]. Based on daily cigarette consumption, current smokers were further categorized as mild (< 10), moderate (11–20), or heavy smokers (> 20) [36].

To estimate alcohol consumption, consumption of alcoholic beverages (various types of wine, beer and spirits) was collected. A visual aid was provided via showing different glass sizes, to help quantify serving sizes. Wine cups or bottles common in daily life or market were provided: 330, 500, 600 ml cups for beer; 15, 25, 100, 125 ml cups for spirits; and 350, 450, 560, 650 ml goblets with scale mark for grape wine. Alcohol content, in grams, was then estimated using standard composition tables. According to drinking status, participants was categorized as non-drinkers, i.e., men who had never drunk alcohol or drank less than once per month in the past 12 months; as current drinkers, i.e., men who drank more than once per month in the past 12 months; or as past drinkers, i.e., who had stopped smoking at least 12 months before the interview [9, 26]. Based on drinking frequency, current drinkers were further categorized as occasional (≤ 2 times/week) and frequent drinkers (≥ 3 times/week). And according to weekly alcohol consumption, current drinkers were categorized as light (< 140 g/week), moderate (140–280 g/week), or heavy drinkers (> 280 g/week) [37].

### Statistical analysis

All statistical analyses were performed using SPSS 25.0 (IBM, Chicago, IL, USA). Normally distributed continuous variables were expressed as mean ± standard error (SE). Categorical variables were expressed as number and percentage (%) and inter-group differences were assessed for significance using the chi-squared test. Adjusted odds ratios (OR) of depressive symptoms and the corresponding 95% confidence interval (CI) according to cigarette or alcohol consumption were estimated by binary logistic regression with forward method, adjusting for confounding factors. Interpolation analysis, using 2nd order Lagrange polynomials, was applied to test for the non-linear dose–effect relationships between the adjusted ORs and the cigarette or alcohol consumption. And ordinal logistic regression was used to evaluate the association of cigarette smoking or alcohol drinking with severity of depressive symptoms adjusting for confounding factors. Analysis of variance for factorial design was applied to examine whether interaction existed between cigarette smoking and alcohol drinking regarding association with depressive symptoms. And it was further evaluated by binary logistic regression, with the product term of cigarette smoking and alcohol drinking added into the model. All *P* values were 2 sided and *P* < 0.05 was considered significant.

## Results

### Participant characteristics

Totally, 6,296 men were recruited from different regions of China: the central (Hubei, *n* = 1151), southern (Guangdong, *n* = 1038), northern (Hebei, *n* = 1093), northeastern (Shanxi, *n* = 1127), southwestern (Guizhou, *n* = 921), and the eastern (Jiangsu, *n* = 966). After excluding 217 who did not fill out questionnaires and 114 who did not provide information on smoking or alcohol exposure, 5,965 men were finally included in the analysis. Characteristics of the participants are shown in Table 1. The average age was 55.93 ± 0.12 years, and men aged 40–49, 50–59, 60–69, 70–79 years were 1,789 (30.0%), 1,977 (33.1%), 1,685 (28.2%), 514 (8.6%), respectively. There are 1,884 (31.6%) never smokers, 3,389 (56.8%) current smokers and 692 (11.6%) past smokers. The distribution of alcohol drinking status was as follows: 1,664 (27.9%) were non-drinkers, 2,188 (36.7%) were occasional drinkers, 1798 (30.1%) were frequent drinkers, and 315 (5.3%) were past drinkers.

**Table 1:** Characteristics of the study population

**Characteristics**	**Total** ***n* = 5,965**	**Depressive symptoms**		*P* value^*^	**Adjusted OR**^a^ **[95% CI]**
		**Yes** ***n* = 1,297**	**No** ***n* = 4,668**		
**Age, yr**	55.93 ± 0.12	55.87 ± 0.14	56.15 ± 0.26	0.352	—
**Age subgroups, yr**					
40–49	1789 (30.0)	378 (21.1)	1411 (78.9)	0.693	—
50–59	1977 (33.1)	423 (21.4)	1554 (78.6)		
60–69	1685 (28.2)	378 (22.4)	137 (77.6)		
70–79	514 (8.6)	118 (23.0)	396 (77.0)		
**Residence**					
Rural	4911(82.3)	1027 (20.9)	3884 (79.1)	0.001	1
City	1054 (17.7)	270 (25.6)	784 (74.4)		0.78 [0.67, 0.92]
**Having spouse**					
Yes	5623 (94.4)	1201 (21.4)	4422 (78.6)	0.004	1
No	335 (5.6)	94 (28.1)	241 (71.9)		0.70 [0.54, 0.90]
**Comorbidity**					
Yes	1556 (26.1)	392 (25.2)	1164 (74.8)	< 0.001	1
No	4409 (73.9)	905 (20.5)	3504 (79.5)		1.29 [1.12, 1.48]
**Cigarette smoking**					
Never	1884 (31.6)	359 (19.1)	1525 (80.9)	—	1
Current	3389 (56.8)	780 (23.0)	2609 (77.0)	0.001	1.32 [1.15, 1.53]
Past	692 (11.6)	158 (22.8)	534 (77.2)	0.034	1.25 [1.01, 1.55]
**Cigarettes/day**					
< 10	747 (22.0)	204 (27.3)	543 (72.7)	0.003	0.84 [0.76, 0.94]
10–20	1300 (38.4)	298 (22.9)	1002 (77.1)		
> 20	1342 (39.6)	278 (20.7)	1064 (79.3)		
**Alcohol drinking**					
Never	1664 (27.9)	350 (21.0)	1314 (79.0)	—	1
Occasional	2188 (36.7)	513 (23.4)	1675 (76.6)	0.075	1.13 [0.97, 1.32]
Frequent	1798 (30.1)	347 (19.3)	1451 (80.7)	0.204	0.86 [0.73, 1.02]
Past	315 (5.3)	87 (27.6)	228 (72.4)	0.010	1.31 [0.99, 1.74]
**Alcohol intake (g/week)**					
< 140	2052 (51.5)	492 (24.0)	1560 (76.0)	< 0.001	0.80 [0.73, 0.88]
140–280	1052 (26.4)	227 (21.6)	825 (78.4)		
> 280	882 (22.1)	141 (16.0)	741 (84.0)		
**Cigarette smoking × ** **alcohol drinking**				0.432	—
**Depression severity**					
Mild	—	567(43.7)	—	—	—
Moderate	—	595(45.9)	—	—	—
Severe	—	135(10.4)	—	—	—

Data are presented as mean ± standard error or as n (%). OR, odds ratio. ^*^P value estimated by chi-squared test

^a^ Odds ratio estimated by binary logistic regression adjusting for age, residence, spouse and comorbidity

There were 1297 (21.7%) participants who scored ≥ 5 in BDI-SF and were considered having depressive symptoms. Among 1,297 men with depressive symptoms, 567 (43.7%), 595 (45.9%) and 135 (10.4%) suffered from mild, moderate, and severe depressive symptoms, respectively. Residing in city (*P* = 0.001), no spouse (*P* = 0.004), and having comorbidity (*P* < 0.001) were associated with higher prevalence of depressive symptoms in middle-aged and elderly men as shown in Table 1.

### Association of cigarette smoking with depressive symptoms

#### Cigarette smoking status

Compared to never smokers (19.1%), the prevalence of depressive symptoms was significantly higher in current smokers (23.0%, *P* = 0.001) and past smokers (22.8%, *P* = 0.034; Table 1). Whereas, no significant difference was observed between current smokers and past smokers (*P* = 0.917). After adjusting for confounding factors including residence, marital status, comorbidity and alcohol drinking, the difference remained. Compared to never smokers, current smokers and past smokers had 1.32-fold (95% CI 1.15–1.53) and 1.25-fold (95% CI 1.01–1.55) odds to suffer from depressive symptoms (Table 1). After stratified by age, the relationship was observed in both the 40–59 and 60–79 age subgroups, except that similar prevalence of depressive symptoms was observed between never and past smokers (OR = 1.06, 95% CI 0.79–1.41) of the 40–59 age subgroup (Additional file 2). However, severity of depressive symptoms was similar among men with different smoking status before (*P* = 0.427) and after (*P* = 0.633) adjusting for confounding factors (Additional file 1).

#### Daily cigarette consumption

**Fig. 1:**
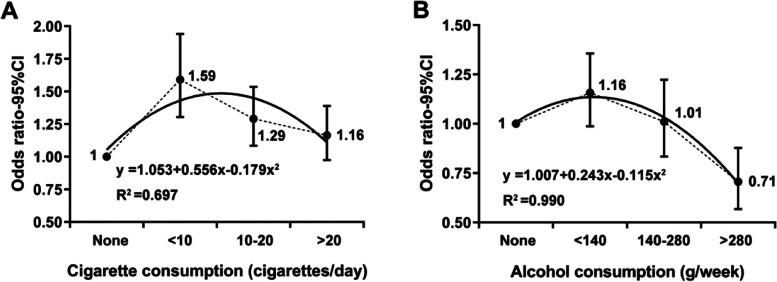
Association between cigarette or alcohol consumption and odds ratio of depressive symptoms. Association between **A** cigarette consumption and **B** alcohol consumption and odds ratio of depressive symptoms. OR, odds ratio; CI, confidence interval. Odds ratio and 95% confidence interval were estimated by binary logistic regression adjusting for residence, spouse and comorbidity. The smoothing fitting curves and the corresponding equations were obtained by interpolation analysis with 2nd order Lagrange polynomials. The dots indicate the adjusted odds ratio of depressive symptoms estimated via binary logistic regression adjusting for residence, spouse and comorbidity. Bars indicate the upper and lower limit of 95% confidence interval of odds ratio

Among 3,389 current smokers, 747 (22.0%) smoked < 10 cigarettes/day, 1,300 (38.4%) smoked 10–20 cigarettes/day, and 1,342 (39.6%) smoked > 20 cigarettes/day (Table 1). Compared to never smokers (19.1%), men who smoked < 10 cigarettes/day (27.3%) and 10–20 cigarettes/day (22.9%) had 59.0% (OR = 1.59, 95% CI 1.30–1.94) and 29.0% (OR = 1.29, 95% CI 1.08–1.54) higher odds of depressive symptoms (Fig. 1A). Prevalence of depressive symptoms tended to be higher although not statistically significant (OR = 1.16, 95% CI 0.98–1.39, *P* = 0.092; Fig. 1A) among men who smoked > 20 cigarettes/day (20.7%), compared to never smokers. The relationship was observed in the 60–79 age subgroup (Additional file 2). For the 40–59 age subgroup, the association pattern remained in general, given that odds of depressive symptoms tended to be higher although not statistically significant in men who smoked < 10 cigarettes/day (OR = 1.28, 95% CI 0.99–1.65) and 10–20 cigarettes/day (OR = 1.23, 95% CI 0.99–1.54), compared to never smokers (Additional file 2). The parabolic-shaped relationship between cigarette consumption and depressive symptoms was shown in Fig. 1A, and may be formulated by the following equation (R^2^ = 0.697, *P* = 0.038): OR = 1.053 + 0.556 × (daily cigarette consumption)-0.179 × (daily cigarette consumption)^2^. Among current smokers, risk of depressive symptoms was negatively associated with daily cigarette consumption (β = -0.17, *P* = 0.001), which was lower by 15.6% (OR = 0.84, 95% CI 0.76–0.94) with daily cigarette consumption increasing by 1 level after adjusting for confounding factors (Table 1).

**Fig. 2:**
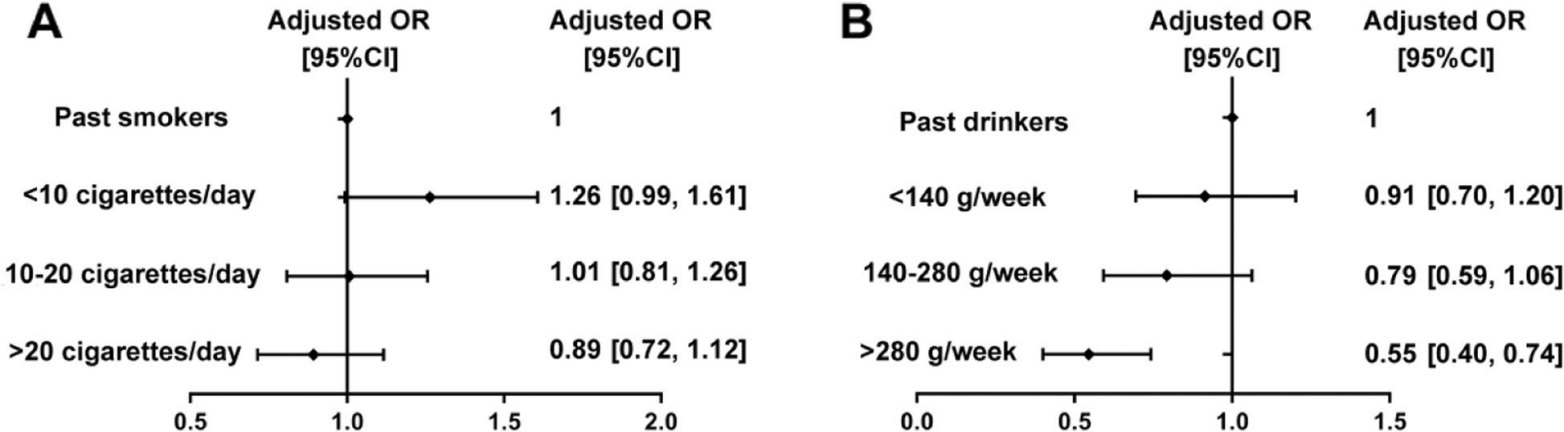
Adjusted odds ratio of depressive symptoms in current smokers or drinkers with various cigarette or alcohol consumption. Adjusted odds ratio of depressive symptoms **A** in current smokers with various cigarette consumption compared to past smokers, and **B** in current drinkers with various alcohol consumption compared to past drinkers. OR, odds ratio; CI, confidence interval. Adjusted odds ratio and 95% confidence interval were estimated by binary logistic regression adjusting for residence, spouse and comorbidity

Compared to past smokers (22.8%), men who smoked < 10 cigarettes/day tended to have higher odd of depressive symptoms, but it was not significant (OR = 1.26, 95% CI 0.99–1.61; Fig. 2A). Whereas, prevalence of depressive symptoms among men who smoked 10–20 cigarettes/day (*P* = 0.950) and > 20 cigarettes/day (*P* = 0.321) were similar to that in past smokers (Fig. 2A). The relationship was observed in both the 40–59 and 60–69 age subgroups (Additional file 2). However, severity of depressive symptoms was similar among smokers with different levels of cigarette consumption without (*P* = 0.373) and with (*P* = 0.845) adjusting for confounding factors (Additional file 1).

### Association of alcohol drinking with depressive symptoms

#### Alcohol drinking status

The prevalence of depressive symptoms was similar among never (21.0%), occasional (23.4%, *P* = 0.075) and frequent drinkers (19.3%, *P* = 0.204; Table 1). The prevalence of depressive symptoms was higher in past drinkers (27.6%, *P* = 0.010), however, it was not significant (OR = 1.31, 95% CI 0.99–1.74, Table 1) after adjusting for confounding factors. The relationship remained generally in both the 40–59 and 60–79 age subgroups, except that lower odds of depressive symptoms was observed in frequent drinkers (OR = 0.72, 95% CI 0.55–0.95) than never drinkers of the 60–79 age subgroup (Additional file 3). Severity of depressive symptoms was not associated with alcohol drinking status before (*P* = 0.143) and after (*P* = 0.246) adjusting for confounding factors (Additional file 1).

#### Weekly alcohol consumption

Among 3,986 current drinkers, 2052 (51.5%) drank < 140 g/week, 1052 (26.4%) drank 140–280 g/week, and 882 (22.1%) drank > 280 g/week (Table 1). Compared to never drinkers (21.0%), those who drank < 140 g/week (24.0%) had higher prevalence of depressive symptoms; however, it became not significant (OR = 1.16, 95% CI 0.99–1.36; Fig. 1B) after adjusting for confounding factors. Prevalence of depressive symptoms was similar between never drinkers and those who drank 140–280 g/week (21.6%, *P* = 0.920; Fig. 1B). Whereas, men who drank > 280 g/week (16.0%) presented 29.4% (OR = 0.71, 95% CI 0.57–0.88; Fig. 1B) lower odds of depressive symptoms than never drinkers. The relationship remained generally in both the 40–59 and 60–79 age subgroups (Additional file 3). The inverted J-shaped relationship between alcohol consumption and prevalence of depressive symptoms was shown in Fig. 1B, and may be formulated by the following equation (R^2^ = 0.990, *P* = 0.033): OR = 1.007 + 0.243 × (weekly alcohol consumption)-0.115 × (weekly alcohol consumption)^2^. Among current drinkers, odds of depressive symptoms were negatively associated with weekly alcohol consumption (β = -0.22, *P* < 0.001), which was lower by 19.9% (OR = 0.80, 95% CI 0.73–0.88) with weekly alcohol consumption increasing by 1 level after adjusting for confounding factors.

Compared to past drinkers (27.6%), similar prevalence of depressive symptoms was observed among men who drank < 140 g/week (24.0%) or 140–280 g/week (21.6%; Fig. 2B). Whereas, odds of depressive symptoms were lower by 45.5% (OR = 0.55, 95% CI 0.40–0.74) among men who drank > 280 g/week (16.0%) than past drinkers (Fig. 2B). And the relationship was observed in both the 40–59 and 60–79 age subgroups (Additional file 3). Severity of depressive symptoms was not associated with weekly alcohol consumption without (*P* = 0.735) and with (*P* = 0.962) adjusting for confounding factors (Additional file 1).

### No interaction was observed between cigarette smoking and alcohol drinking regarding depressive symptoms

Participants were categorized into 12 groups according to cigarette smoking status (3 levels) and alcohol drinking status (4 levels). Analysis of variance for the factorial design was applied to compare the scores of BDI-SF among the fully-crossed groups. The interactive effect of cigarette smoking and alcohol drinking was not significant (*P* = 0.962). It was further explored by adding the product term of cigarette smoking and alcohol drinking into the binary logistic regression model with the existence of depressive symptoms as the dependent variable, and the product term was not significant (*P* = 0.432, Table 1). It showed that despite associations of depressive symptoms with cigarette smoking and alcohol drinking, they were not interacted.

## Discussion

This is the first large-scale cross-sectional study evaluating the dose–response associations of cigarette and alcohol consumption with depressive symptoms among middle-aged and elderly men. A parabolic-shaped relationship was observed between cigarette consumption and depressive symptoms. Compared to non-smokers, higher odds of depressive symptoms were observed in light and mild smokers, whereas similar odds in heavy smokers. An inverted J-shaped relationship was observed between alcohol consumption and depressive symptoms. Compared to non-drinkers, higher risk of depressive symptoms was observed in light drinkers, and similar odds in mild drinkers, whereas significantly lower odds in heavy drinkers. Among current smokers, prevalence of depressive symptoms was negatively associated with cigarette consumption, and a negative pattern was also observed in current drinkers. These findings suggest that association between depressive symptoms and cigarette or alcohol differed with consumption. Relief of depressive symptoms may be one of the important motivations for smokers or drinkers to increase cigarette or alcohol consumption. Health-care providers should exercise great caution on depressive mood of men in conducting cigarette and alcohol prevention and control. In addition, neither cigarette nor alcohol cessation was associated with lower prevalence of depressive symptoms than current smoking and drinking, especially when compared with heavy smoking and drinking. This finding implies that depressive mood may constitute an impediment to cigarette or alcohol cessation and therefore should be considered in conducting cessation treatment. Whereas, no interaction was observed between cigarette smoking and alcohol drinking regarding associations with depressive symptoms.

Consistent with majority previous studies in Western high-income countries [4–6], higher prevalence of depressive symptoms was found among current smokers in this study. Accordingly, it seems reasonable to infer that higher daily cigarette consumption is associated with higher odds of depressive symptoms. However, contrary to our expectation, daily cigarette consumption was found to be negatively associated with depressive symptoms among current smokers in this study. The parabolic-shaped relationship between cigarette consumption and depressive symptoms implies that cigarette may have both beneficial and adverse effects on depressive symptoms. Cigarette may lead to depressive symptoms via acting on an individual’s neurocircuitry and increasing susceptibility to environmental stress [6, 15]. On the other hand, cigarette as a psychoactive substance can change neural activity after intake and provide a boost to mood [38–40]. These psychoactive effects of cigarette increase with consumption in a certain range [40]. Higher smoking amount was found to be associated with stronger inhibiting effect on human brain punishing center [40, 41]. Therefore, smokers with higher cigarette consumption present lower risk of negative sense of the self and self-esteem which is resistant to depressive symptoms.

Significantly higher prevalence of depressive symptoms was observed in men who smoked < 10 and 10–20 cigarettes/day than non-smokers. This may reflect that, in low- and medium-dose exposure of cigarette, the development of smoking-related depressive symptoms may overpower any of its protective effects. Whereas, in high-dose exposure, the benefits of smoking appear to balance its hazards, and similar prevalence of depressive symptoms was observed between men who smoked > 20 cigarettes/day and non-smokers. This is consistent with a prospective study among Chinese middle-aged and elderly participants, in which similar incidence or persistence of depressive symptoms was observed between heavy smokers and non-smokers [31]. The negative association between cigarette consumption and depressive symptoms seems to support the psycho-relaxant effects of cigarette, and implies that relief of depressive symptoms may be one of the important motivations for smokers to increase cigarette consumption.

The majority of existing evidences have demonstrated a J-shaped relationship between alcohol consumption and depressive symptoms, i.e., beneficial effects of light or moderate drinking and adverse effects of heavy drinking on depressive symptoms [11–13]. Whereas, a reversed pattern was observed in this study, i.e., higher risk of depressive symptoms associated with light drinking and a lower risk with heavy drinking among Chinese middle-aged and elderly men. Genetic and cultural factors may partly account for the controversy. Firstly, Han Chinese population has high frequencies of genetic polymorphism encoding alcohol dehydrogenase and aldehyde dehydrogenase which produce an instantaneous unpleasant flushing effect after alcohol intake [22, 23, 26]. It prevents a substantial proportion of Chinese from developing into alcohol use disorder and induces a moderate drinking pattern among Chinese [22, 25]. Secondly, in China, alcoholic beverage acts as a catalyst linking together families, friends, and guests, helping to establish, develop, and consolidate solidarity among them [21, 26]. Therefore, Chinese show a preference for drinking at meals with families or in social occasions, distinct from the drinking pattern in Western high-income countries which value uniqueness of individuality [21, 22, 26].

There are several possible explanations for higher risk of depressive symptoms with light drinking. Firstly, depressive symptoms typically result in self-imposed social isolation at which time the need to meet the social demand for collective drinking will be reduced [31]. Secondly, according to the self-medication theory, individuals drink alcohol to alleviate depressive symptoms [40]. The mechanisms underlying the lower odds of depressive symptoms with heavy drinking remains unclear, but it may be due to the followings. Higher alcohol amount was found to be associated with stronger stimulative effect on human brain rewarding center [40, 41], which may partly account for the lower risk of depressive symptoms with heavy drinking. Accordingly, drinkers with higher alcohol consumption tend to have more positive sense of the self-esteem, which is resistant to depressive symptoms. Besides, higher alcohol consumption in Chinese may represent a closer family relationship and a better social interaction [21, 22, 26]. They contribute to the prevention and relief of depressive symptoms and are possible mechanisms underlying the significantly lower prevalence of depressive symptoms among men drinking > 280 g/week.

Different from majority previous studies [4, 42–44], smoking cessation was not associated with lower odds of depressive symptoms than current smoking. Smoking abstainers presented similar prevalence of depressive symptoms to current smokers. According to the negative relationship between cigarette consumption and depressive symptoms in this study, past smokers presented a tendency of lower odds of depressive symptoms than light smokers, similar odds to mild and heavy smokers. Therefore, the proportions of light, mild and heavy smokers in the study population may affect the conclusions. Vast majority (73%) men in this study were mild and heavy smokers, which may contribute to the similar prevalence of depressive symptoms between past smokers and current smokers. Contrary to previous studies [31, 42], alcohol cessation tended to be associated with higher prevalence of depressive symptoms in this study. Given the critical role of alcoholic beverage in familiar and social linkage, the alcohol cessation-induced familial and social isolation may contribute to higher prevalence of depressive symptoms among alcohol abstainers [21, 26]. This finding implies that depressive mood may constitute an impediment to cigarette or alcohol cessation.

There are several limitations for this study. Firstly, the cross-sectional design of this study precludes us from determining the causal and sequential associations between cigarette or alcohol and depressive symptoms. It’s difficult to determine whether vulnerability of depressive symptoms increases risk for progression to cigarette smoking or vice versa. Secondly, the measurement of depressive symptoms was based on a non-diagnostic, self-reported scale. Despite good validity and reliability of BDI-SF in Chinese population, it is only a screening for depressive symptoms but not a clinical diagnosis of depression. Besides, it is possible that the observed associations could result from unmeasured confounding effects of other lifestyle factors such as diet, sleep, exercise et al. Finally, the gender difference in the underlying mechanisms, patterns, and courses of smoking and drinking behaviors limit extension of the findings in this study to the women [31]. Future examination of other health behaviors in models of depression would be useful.

## Conclusion

The associations of cigarette smoking and alcohol drinking with depressive symptoms differ with consumption in middle-aged and elderly men. A parabolic-shaped relationship was observed between cigarette consumption and depressive symptoms. And an inverted J-shaped relationship was observed between alcohol consumption and depressive symptoms. Relief of depressive symptoms may be an important motivation for men to increase their cigarette or alcohol consumption. And health-care providers should exercise great caution on depressive symptoms in conducting cigarette and alcohol control.

## Supplementary Information

**Additional file 1.** Depression severity according to status and consumption of cigarette smoking or alcohol drinking.

**Additional file 2.** Odds of depression symptoms according to status and consumption of cigarette smoking stratified by age.

**Additional file 3.** Odds of depression symptoms according to status and consumption of alcohol drinking stratified by age.

## Data Availability

The datasets used and/or analyzed during the current study are available from the corresponding author on reasonable request.

## Abbreviations

BDI-SF: Beck Depression Inventory-Short Form
SE: Standard error
OR: Odds ratio
CI: Confidence interval

## References

[1] Comijs HC, Nieuwesteeg J, Kok R, van Marwijk HW, van der Mast RC, Naarding P (2015). The two-year course of late-life depression; results from the Netherlands study of depression in older persons. BMC Psychiatry.

[2] Reynolds K, Pietrzak RH, El-Gabalawy R, Mackenzie CS, Sareen J (2015). Prevalence of psychiatric disorders in U.S. older adults: findings from a nationally representative survey. World Psychiatry..

[3] Conwell Y, Van Orden K, Caine ED (2011). Suicide in older adults. Psychiatr Clin North Am..

[4] Bakhshaie J, Zvolensky MJ, Goodwin RD (2015). Cigarette smoking and the onset and persistence of depression among adults in the United States: 1994–2005. Compr Psychiatry.

[5] King JL, Reboussin BA, Spangler J, Cornacchione Ross J, Sutfin EL (2018). Tobacco product use and mental health status among young adults. Addict Behav.

[6] Ranjit A, Korhonen T, Buchwald J, Heikkila K, Tuulio-Henriksson A, Rose RJ (2019). Testing the reciprocal association between smoking and depressive symptoms from adolescence to adulthood: a longitudinal twin study. Drug Alcohol Depend.

[7] Bellos S, Skapinakis P, Rai D, Zitko P, Araya R, Lewis G (2016). Longitudinal association between different levels of alcohol consumption and a new onset of depression and generalized anxiety disorder: results from an international study in primary care. Psychiatry Res.

[8] Gea A, Beunza JJ, Estruch R, Sanchez-Villegas A, Salas-Salvado J, Buil-Cosiales P (2013). Alcohol intake, wine consumption and the development of depression: the PREDIMED study. BMC Med.

[9] Gemes K, Forsell Y, Janszky I, Laszlo KD, Lundin A, Ponce De Leon A (2019). Moderate alcohol consumption and depression - a longitudinal population-based study in Sweden. Acta Psychiatr Scand..

[10] Garcia-Esquinas E, Ortola R, Galan I, Soler-Vila H, Laclaustra M, Rodriguez-Artalejo F (2018). Moderate alcohol drinking is not associated with risk of depression in older adults. Sci Rep.

[11] An R, Xiang X (2015). Smoking, heavy drinking, and depression among U.S. middle-aged and older adults. Prev Med..

[12] Boden JM, Fergusson DM (2011). Alcohol and depression. Addiction.

[13] Sullivan LE, Fiellin DA, O'Connor PG (2005). The prevalence and impact of alcohol problems in major depression: a systematic review. Am J Med.

[14] Castro Y, Costello TJ, Correa-Fernandez V, Heppner WL, Reitzel LR, Cofta-Woerpel L (2011). Differential effects of depression on smoking cessation in a diverse sample of smokers in treatment. Am J Prev Med.

[15] Fluharty M, Taylor AE, Grabski M, Munafo MR (2017). The association of cigarette smoking with depression and anxiety: a systematic review. Nicotine Tob Res.

[16] Weinberger AH, Kashan RS, Shpigel DM, Esan H, Taha F, Lee CJ (2017). Depression and cigarette smoking behavior: a critical review of population-based studies. Am J Drug Alcohol Abuse.

[17] Abulseoud OA, Karpyak VM, Schneekloth T, Hall-Flavin DK, Loukianova LL, Geske JR (2013). A retrospective study of gender differences in depressive symptoms and risk of relapse in patients with alcohol dependence. Am J Addict.

[18] Boykoff N, Schneekloth TD, Hall-Flavin D, Loukianova L, Karpyak VM, Stevens SR (2010). Gender differences in the relationship between depressive symptoms and cravings in alcoholism. Am J Addict.

[19] Husky MM, Mazure CM, Paliwal P, McKee SA (2008). Gender differences in the comorbidity of smoking behavior and major depression. Drug Alcohol Depend.

[20] Karpyak VM, Geske JR, Hall-Flavin DK, Loukianova LL, Schneekloth TD, Skime MK (2019). Sex-specific association of depressive disorder and transient emotional states with alcohol consumption in male and female alcoholics. Drug Alcohol Depend.

[21] Jiang L (2011). Comparison of the difference between Chinese and Western drinking culture. Asian Social Science..

[22] Luczak SE, Wall TL, Shea SH, Byun SM, Carr LG (2001). Binge drinking in Chinese, Korean, and White college students: genetic and ethnic group differences. Psychol Addict Behav.

[23] Rueger SY, Hu H, McNamara P, Cao D, Hao W, King AC (2015). Differences in subjective response to alcohol in heavy- and light-drinking Chinese men versus Caucasian American men. Addiction.

[24] Cheng H, Lee S, Tsang A, Huang Y, Liu Z, Anthony JC (2010). The epidemiological profile of alcohol and other drug use in metropolitan China. Int J Public Health.

[25] Cheng HG, Deng F, Xiong W, Phillips MR (2015). Prevalence of alcohol use disorders in mainland China: a systematic review. Addiction.

[26] Cochrane J, Chen H, Conigrave KM, Hao W (2003). Alcohol use in China. Alcohol Alcohol.

[27] Audrain-McGovern J, Rodriguez D, Rodgers K, Cuevas J (2011). Declining alternative reinforcers link depression to young adult smoking. Addiction.

[28] Goodwin RD, Perkonigg A, Hofler M, Wittchen HU (2013). Mental disorders and smoking trajectories: a 10-year prospective study among adolescents and young adults in the community. Drug Alcohol Depend.

[29] McKenzie M, Olsson CA, Jorm AF, Romaniuk H, Patton GC (2010). Association of adolescent symptoms of depression and anxiety with daily smoking and nicotine dependence in young adulthood: findings from a 10-year longitudinal study. Addiction.

[30] Sihvola E, Rose RJ, Dick DM, Pulkkinen L, Marttunen M, Kaprio J (2008). Early-onset depressive disorders predict the use of addictive substances in adolescence: a prospective study of adolescent Finnish twins. Addiction.

[31] Cheng HG, Chen S, McBride O, Phillips MR (2016). Prospective relationship of depressive symptoms, drinking, and tobacco smoking among middle-aged and elderly community-dwelling adults: Results from the China Health and Retirement Longitudinal Study (CHARLS). J Affect Disord.

[32] Liu Q, Peng X, Gu Y, Shang X, Zhou Y, Zhang H (2021). Associations between smoking, sex hormone levels and late-onset hypogonadism in men differ depending on age. Aging (Albany NY).

[33] Bao YP, Qiu Y, Yan SY, Jia ZJ, Li SX, Lian Z (2013). Pattern of drug use and depressive symptoms among amphetamine type stimulants users in Beijing and Guangdong province, China. PLoS ONE.

[34] Shek DTL (1990). Reliability and factorial structure of the chinese version of the Beck Depression Inventory. J Clin Psychol.

[35] Han T, Zhang S, Duan W, Ren X, Wei C, Sun C (2019). Eighteen-year alcohol consumption trajectories and their association with risk of type 2 diabetes and its related factors: the China Health and Nutrition Survey. Diabetologia.

[36] Svartberg J, Jorde R (2007). Endogenous testosterone levels and smoking in men the fifth Tromso study. Int J Androl..

[37] The Dietary Guidelines for Chinese Residents. http://dg.cnsoc.org/. Accessed 31 Jul 2022.

[38] Audrain-McGovern J, Rodriguez D, Kassel JD (2009). Adolescent smoking and depression: evidence for self-medication and peer smoking mediation. Addiction.

[39] Chaiton M, Cohen J, O'Loughlin J, Rehm J (2010). Use of cigarettes to improve affect and depressive symptoms in a longitudinal study of adolescents. Addict Behav.

[40] Cheng W, Rolls ET, Robbins TW, Gong W, Liu Z, Lv W (2019). Decreased brain connectivity in smoking contrasts with increased connectivity in drinking. Elife..

[41] Cheng W, Rolls ET, Qiu J, Liu W, Tang Y, Huang CC (2016). Medial reward and lateral non-reward orbitofrontal cortex circuits change in opposite directions in depression. Brain.

[42] Lechner WV, Sidhu NK, Cioe PA, Kahler CW (2019). Effects of time-varying changes in tobacco and alcohol use on depressive symptoms following pharmaco-behavioral treatment for smoking and heavy drinking. Drug Alcohol Depend.

[43] Amiri S (2021). The prevalence of depression symptoms after smoking cessation: a systematic review and meta-analysis. J Addict Dis.

[44] Taylor G, McNeill A, Girling A, Farley A, Lindson-Hawley N, Aveyard P (2014). Change in mental health after smoking cessation: systematic review and meta-analysis. BMJ.

