# Supplementary material for: The dose–effect relationships of cigarette and alcohol consumption with depressive symptoms: a multiple-center, cross-sectional study in 5965 Chinese middle-aged and elderly men
Source: BMC Psychiatry. 2022 Oct 25;22:657. doi: 10.1186/s12888-022-04316-0 (PMC9594935; doi:10.1186/s12888-022-04316-0)
Supplement: Supplementary file 1 — Additional file 1. Depression severity according to status and consumption of cigarette smoking or alcohol drinking. [file 12888_2022_4316_MOESM1_ESM.docx]

**Additional file 1. Depression severity according to status and consumption of cigarette smoking or alcohol drinking**.

|  | **Depression severity** | |  |  |
| --- | --- | --- | --- | --- |
|  | **Light** | **Moderate or severe** | ***P* value**^*^ | **Adjusted *P* value** ^Ɨ^ |
| **Cigarette smoking** |  |  |  |  |
| Never | 147 (40.9) | 212 (59.1) | 0.427 | 0.633 |
| Current | 347 (44.5) | 433 (55.5) |  |  |
| Past | 73 (46.2) | 85 (53.8) |  |  |
| **Cigarettes/day** |  |  |  |  |
| <10 | 86 (42.2) | 118 (57.8) | 0.373 | 0.845 |
| 10-20 | 142 (47.7) | 156 (52.3) |  |  |
| >20 | 119 (42.8) | 159 (57.2) |  |  |
| **Alcohol Drinking** |  |  |  |  |
| Never | 139 (39.7) | 211 (60.3) | 0.143 | 0.246 |
| Occasional | 227 (44.2) | 286 (55.8) |  |  |
| Frequent | 155 (44.7) | 192 (55.3) |  |  |
| Past | 46 (52.9) | 41 (47.1) |  |  |
| **Alcohol intake (g/week)** |  |  |  |  |
| <140 | 213 (43.3) | 279 (56.7) | 0.735 | 0.962 |
| 140-280 | 105 (46.3) | 122 (53.7) |  |  |
| >280 | 64 (45.4) | 77 (54.6) |  |  |

Data are presented as n (%). ^*^*P* value estimated by chi-squared test. ^Ɨ^*P* value estimated by binary logistic regression adjusting for age, residence, spouse and comorbidity.
