# Supplementary material for: The dose–effect relationships of cigarette and alcohol consumption with depressive symptoms: a multiple-center, cross-sectional study in 5965 Chinese middle-aged and elderly men
Source: BMC Psychiatry. 2022 Oct 25;22:657. doi: 10.1186/s12888-022-04316-0 (PMC9594935; doi:10.1186/s12888-022-04316-0)
Supplement: Supplementary file 2 — Additional file 2. Odds of depression symptoms according to status and consumption of cigarette smoking stratified by age. [file 12888_2022_4316_MOESM2_ESM.docx]

**Additional file 2. Odds of depression symptoms according to status and consumption of cigarette smoking stratified by age**.

|  | **Men aged 40-59 years** | | | **Men aged 60-79 years** | | |
| --- | --- | --- | --- | --- | --- | --- |
| **Characteristics** | **Depressive symptoms, n (%)** | **OR**^Ɨ^ **[95% CI]**  **versus never smokers** | **OR**^Ɨ^ **[95% CI] versus past smokers** | **Depressive symptoms, n (%)** | **OR**^Ɨ^ **[95% CI] versus never smokers** | **OR**^Ɨ^ **[95% CI] versus past smokers** |
| **Cigarette smoking** |  |  |  |  |  |  |
| Never | 237 (19.8) | 1 | — | 122 (17.7) | 1 | — |
| Current | 485 (22.1) | 1.20 [1.00, 1.43]^*^ | — | 295 (24.6) | 1.60 [1.25, 2.04]^***^ | — |
| Past | 79 (20.7) | 1.06 [0.79, 1.41] | — | 79 (25.4) | 1.63 [1.16, 2.28] ^**^ | — |
| **Cigarettes/day** |  |  |  |  |  |  |
| <10 | 117 (24.4) | 1.28 [0.99, 1.65] | 1.21 [0.88, 1.68] | 87 (32.5) | 2.21 [1.60, 3.05] ^***^ | 1.39 [0.97, 2.00] |
| 10-20 | 191 (22.8) | 1.23 [0.99, 1.54] | 1.14 [0.84, 1.53] | 107 (23.1) | 1.40 [1.04, 1.87] ^*^ | 0.88 [0.63, 1.23] |
| >20 | 177 (20.2) | 1.11 [0.89, 1.39] | 0.97 [0.72, 1.31] | 101 (21.7) | 1.29 [0.96, 1.73] | 0.81 [0.58, 1.14] |

OR, odds ratio. ^Ɨ^Odds ratio estimated by binary logistic regression adjusting for age, residence, spouse and comorbidity. ^*^*P*<0.05, ^**^ *P*<0.01, ^***^ *P*<0.001.
