# Supplementary material for: The dose–effect relationships of cigarette and alcohol consumption with depressive symptoms: a multiple-center, cross-sectional study in 5965 Chinese middle-aged and elderly men
Source: BMC Psychiatry. 2022 Oct 25;22:657. doi: 10.1186/s12888-022-04316-0 (PMC9594935; doi:10.1186/s12888-022-04316-0)
Supplement: Supplementary file 3 — Additional file 3. Odds of depression symptoms according to status and consumption of alcohol drinking stratified by age. [file 12888_2022_4316_MOESM3_ESM.docx]

|  | **Men aged 40-59 years** | | | **Men aged 60-79 years** | | |
| --- | --- | --- | --- | --- | --- | --- |
| **Characteristics** | **Depressive symptoms, n (%)** | **OR**^Ɨ^ **[95% CI]**  **versus never drinkers** | **OR**^Ɨ^ **[95% CI] versus past drinkers** | **Depressive symptoms, n (%)** | **OR**^Ɨ^ **[95% CI] versus never drinkers** | **OR**^Ɨ^ **[95% CI] versus past drinkers** |
| **Alcohol drinking** |  |  |  |  |  |  |
| Never | 168 (19.6) | 1 | — | 182 (22.5) | 1 | — |
| Occasional | 363 (23.0) | 1.21 [0.98, 1.49] | — | 150 (24.6) | 1.04 [0.81, 1.34] | — |
| Frequent | 229 (19.2) | 0.97 [0.78, 1.22] | — | 118 (19.4) | 0.72 [0.55, 0.95] ^*^ | — |
| Past | 41 (28.9) | 1.60 [1.07, 2.40] ^*^ | — | 46 (26.6) | 1.04 [0.70, 1.54] | — |
| **Alcohol intake (g/week)** |  |  |  |  |  |  |
| <140 | 347 (23.6) | 1.24 [1.00, 1.52] ^*^ | 0.81 [0.55, 1.19] | 145 (24.8) | 1.05 [0.81, 1.35] | 1.05 [0.71, 1.57] |
| 140-280 | 152 (21.2) | 1.08 [0.85, 1.39] | 0.71 [0.47, 1.07] | 75 (22.5) | 0.88 [0.65, 1.21] | 0.87 [0.56, 1.34] |
| >280 | 93 (16.0) | 0.78 [0.59, 1.03] | 0.50 [0.32, 0.76] ^**^ | 48 (16.1) | 0.58 [0.40, 0.83] ^**^ | 0.57 [0.35, 0.90] ^*^ |

**Additional file 3. Odds of depression symptoms according to status and consumption of alcohol drinking stratified by age**.

OR, odds ratio. ^Ɨ^Odds ratio estimated by binary logistic regression adjusting for age, residence, spouse and comorbidity. ^*^*P*<0.05, ^**^ *P*<0.01.
